# Representation and Misrepresentation of Scientific Evidence in Contemporary Tobacco Regulation: A Review of Tobacco Industry Submissions to the UK Government Consultation on Standardised Packaging

**DOI:** 10.1371/journal.pmed.1001629

**Published:** 2014-03-25

**Authors:** Selda Ulucanlar, Gary J. Fooks, Jenny L. Hatchard, Anna B. Gilmore

**Affiliations:** 1 University of Bath, Bath, United Kingdom; 2 UK Centre for Alcohol and Tobacco Studies (UKCTAS), University of Bath, Bath, United Kingdom; University of Queensland, Australia

## Abstract

**Background:**

Standardised packaging (SP) of tobacco products is an innovative tobacco control measure opposed by transnational tobacco companies (TTCs) whose responses to the UK government's public consultation on SP argued that evidence was inadequate to support implementing the measure. The government's initial decision, announced 11 months after the consultation closed, was to wait for ‘more evidence’, but four months later a second ‘independent review’ was launched. In view of the centrality of evidence to debates over SP and TTCs' history of denying harms and manufacturing uncertainty about scientific evidence, we analysed their submissions to examine how they used evidence to oppose SP.

**Methods and Findings:**

We purposively selected and analysed two TTC submissions using a verification-oriented cross-documentary method to ascertain how published studies were used and interpretive analysis with a constructivist grounded theory approach to examine the conceptual significance of TTC critiques. The companies' overall argument was that the SP evidence base was seriously flawed and did not warrant the introduction of SP. However, this argument was underpinned by three complementary techniques that misrepresented the evidence base. First, published studies were repeatedly misquoted, distorting the main messages. Second, ‘mimicked scientific critique’ was used to undermine evidence; this form of critique insisted on methodological perfection, rejected methodological pluralism, adopted a litigation (not scientific) model, and was not rigorous. Third, TTCs engaged in ‘evidential landscaping’, promoting a parallel evidence base to deflect attention from SP and excluding company-held evidence relevant to SP. The study's sample was limited to sub-sections of two out of four submissions, but leaked industry documents suggest at least one other company used a similar approach.

**Conclusions:**

The TTCs' claim that SP will not lead to public health benefits is largely without foundation. The tools of Better Regulation, particularly stakeholder consultation, provide an opportunity for highly resourced corporations to slow, weaken, or prevent public health policies.

*Please see later in the article for the Editors' Summary*

## Abstract 2

Selda Ulucanlar and colleagues analyze submissions by two tobacco companies to the UK government consultation on standardized packaging.

*Please see later in the article for the Editors' Summary*

## Editors' Summary

**Background:**

Every year, about 6 million people die from tobacco-related diseases and, if current trends continue, annual tobacco-related deaths will increase to more than 8 million by 2030. To reduce this loss of life, national and international bodies have drawn up various conventions and directives designed to implement tobacco control measures such as the adoption of taxation policies aimed at reducing tobacco consumption and bans on tobacco advertising, promotion, and sponsorship. One innovative but largely unused tobacco control measure is standardised packaging of tobacco products. Standardised packaging aims to prevent the use of packaging as a marketing tool by removing all brand imagery and text (other than name) and by introducing packs of a standard shape and colour that include prominent pictorial health warnings. Standardised packaging was first suggested as a tobacco control measure in 1986 but has been consistently opposed by the tobacco industry.

**Why Was This Study Done?:**

The UK is currently considering standardised packaging of tobacco products. In the UK, Better Regulation guidance obliges officials to seek the views of stakeholders, including corporations, on the government's cost and benefit estimates of regulatory measures such as standardised packaging and on the evidence underlying these estimates. In response to a public consultation about standardised packaging in July 2013, which considered submissions from several transnational tobacco companies (TTCs), the UK government announced that it would wait for the results of the standardised packaging legislation that Australia adopted in December 2012 before making its final decision about this tobacco control measure. Parliamentary debates and media statements have suggested that doubt over the adequacy of the evidence was the main reason for this ‘wait and see’ decision. Notably, TTCs have a history of manufacturing uncertainty about the scientific evidence related to the harms of tobacco. Given the centrality of evidence to the debate about standardised packaging, in this study, the researchers analyse submissions made by two TTCs, British American Tobacco (BAT) and Japan Tobacco International (JTI), to the first UK consultation on standardised packaging (a second review is currently underway and will report shortly) to examine how TTCs used evidence to oppose standardised packaging.

**What Did the Researchers Do and Find?:**

The researchers analysed sub-sections of two of the four TTC submissions (those submitted by BAT and JTI) made to the public consultation using verification-oriented cross-documentary analysis, which compared references made to published sources with the original sources to ascertain how these sources had been used, and interpretative analysis to examine the conceptual significance of TTC critiques of the evidence on standardised packaging. The researchers report that the companies' overall argument was that the evidence base in support of standardised packaging was seriously flawed and did not warrant the introduction of such packaging. The researchers identified three ways in which the TTC reports misrepresented the evidence base. First, the TTCs misquoted published studies, thereby distorting the main messages of these studies. For example, the TTCs sometimes omitted important qualifying information when quoting from published studies. Second, the TTCs undermined evidence by employing experts to review published studies for methodological rigor and value in ways that did not conform to normal scientific critique approaches (‘mimicked scientific critique’). So, for example, the experts considered each piece of evidence in isolation for its ability to support standardised packaging rather than considering the cumulative weight of the evidence. Finally, the TTCs engaged in ‘evidential landscaping’. That is, they promoted research that deflected attention from standardised packaging (for example, research into social explanations of smoking behaviour) and omitted internal industry research on the role of packaging in marketing.

**What Do These Findings Mean?:**

These findings suggest that the TTC critique of the evidence in favour of standardised packaging that was presented to the UK public consultation on this tobacco control measure is highly misleading. However, because the researchers' analysis only considered subsections of the submissions from two TTCs, these findings may not be applicable to the other submissions or to other TTCs. Moreover, their analysis only considered the efforts made by TTCs to influence public health policy and not the effectiveness of these efforts. Nevertheless, these findings suggest that the claim of TTCs that standardised packaging will not lead to public health benefits is largely without foundation. More generally, these findings highlight the possibility that the tools of Better Regulation, particularly stakeholder consultation, provide an opportunity for wealthy corporations to slow, weaken, or prevent the implementation of public health policies.

**Additional Information:**

Please access these websites via the online version of this summary at http://dx.doi.org/10.1371/journal.pmed.1001629.

- The World Health Organization provides information about the dangers of tobacco (in several languages) and an article about first experiences with Australia's tobacco plain packaging law; for information about the tobacco industry's influence on policy, see the 2009 World Health Organization report ‘Tobacco industry interference with tobacco control’
- A UK parliamentary briefing on standardised packaging of tobacco products, a press release about the consultation, and a summary report of the consultation are available; the ideas behind the UK's Better Regulation guidance are described in a leaflet produced by the Better Regulation Task Force
- Cancer Research UK (CRUK) has a web page with information on standardised packaging and includes videos
- Wikipedia has a page on standardised packaging of tobacco products (note: Wikipedia is a free online encyclopaedia that anyone can edit; available in several languages)
- The UK Centre for Tobacco Control Studies is a network of UK universities that undertakes original research, policy development, advocacy, and teaching and training in the field of tobacco control
- TobaccoTactics.org, an online resource managed by the University of Bath, provides up-to-date information on the tobacco industry and the tactics it uses to influence tobacco regulation
- SmokeFree, a website provided by the UK National Health Service, offers advice on quitting smoking and includes personal stories from people who have stopped smoking
- Smokefree.gov, from the US National Cancer Institute, offers online tools and resources to help people quit smoking

## Introduction

Beginning in the mid-1990s, governments globally have sought to transform policymaking and regulatory activity through a number of reforms collectively known in Europe as Better Regulation [1]. Although these reforms are expected to make policymaking more transparent and evidence-based, in some jurisdictions their implementation has been shown to occur under pressure from corporations whose products are damaging to health, including British American Tobacco (BAT), with the expectation that the associated procedures would make it harder to pass public health policies [2]. In the UK, Better Regulation is underpinned by neoliberal assumptions concerning business competitiveness, and official guidance declares that regulation should not ‘impose costs and obligations’ on business and other groups ‘unless a robust and compelling case has been made’ [3]. This process in turn requires the methodical identification and valuation of all potential costs and benefits of proposed regulation, achieved predominantly through impact assessment and stakeholder consultation [4]. Officials are obliged to seek the views of stakeholders, including corporations, on the government's cost and benefit estimates as well as underlying ‘key assumptions and data’ [3]. Commercial entities that will be affected by proposed regulation are thus given an explicit role in evaluating, confirming, or disputing evidence used in formulating those policies.

The requirements of Better Regulation have significant implications for public health, where major corporations with products damaging to health, including transnational tobacco companies (TTCs) and alcohol [5] and food [6],[7] industries, have a long track record (in tobacco's case, dating back to at least the early 1950s) of manipulating and misrepresenting evidence to deny the harms of their products, question the benefits and highlight the costs of public health policies [8]. TTCs have systematically sought to fabricate doubt and controversy over evidence unfavourable to their interests by labelling research demonstrating tobacco's harms as ‘junk science’, commissioning their own research to challenge established evidence on tobacco's harms or policy impacts [9],[10], and promoting a set of industry-specified scientific standards collectively termed ‘sound science’ and ‘good epidemiology practices’ [11],[12]. This evidence of TTC willingness to challenge and undermine the scientific evidence on tobacco harms and on policy impacts [12],[13],[14],[15],[16],[17],[18],[19],[20],[21] raises concerns that stakeholder consultation and impact assessment may make it easier for TTCs to successfully obstruct public health policies and poses a challenge to the underlying assumption of Better Regulation that evidence optimises policymaking, rendering it more rational and efficient.

The UK experience with standardised packaging (SP) of tobacco products provides a major opportunity to explore the tobacco industry's current approach to and use of evidence. SP aims to prevent the use of packaging as a powerful marketing tool by removing all brand imagery and text (other than name) and instead implementing standard shape/colour packs with prominent pictorial health warnings. First mooted in 1986, SP was consistently opposed by the tobacco industry [22] and it was only in December 2012 that Australia became the first country to adopt the policy [23], despite threatened and ongoing litigation by TTCs [24],[25]. The UK was the next country to consider SP formally; following a four-month public consultation and 11 months of deliberation, the government announced that it had decided to ‘wait and see’ how the Australian experience ‘panned out’ before making a decision [26]. Parliamentary debates [26] and media statements indicated that doubts over the adequacy of the evidence (and the related anticipation of legal challenge by TTCs) were the main reason for the government's hesitance [27],[28]. However, four months later, when it became clear that SP might be introduced through House of Lords amendments to unrelated legislation on children and families, the government announced a further, independent review of existing and new evidence on SP that is to report in March 2014 [29].

Prior to the consultation, the Department of Health (DH) commissioned a systematic review of the evidence base on SP (hereafter referred to as the Moodie review, after the lead author's name), which concluded that there was ‘strong evidence’ that SP would reduce the attractiveness and appeal of tobacco products and increase the effectiveness of health warnings and, therefore, had the potential to ‘contribute to reductions in the harm caused by tobacco smoking’ [30]. The four main TTCs (BAT, Japan Tobacco International (JTI), Philip Morris Limited (PM), and Imperial Tobacco) submitted large volumes of written submissions to the stakeholder consultation. Our initial analysis of these showed that the companies' main collective argument was that SP would not ‘work’ [31]. The TTCs, on the one hand, dismissed the existing evidence base indicating a likely positive impact of SP and, on the other hand, cited a large volume of alternative evidence (77 items) to support their argument that SP would not work. However, only a small number (17) of this alternative body of evidence was directly relevant to SP, and of these, the majority (14) was industry funded/linked and none was peer-reviewed [31]. By contrast, the studies included in the Moodie review were all relevant, the majority were peer-reviewed, and none was industry funded/linked.

Here, we build on this initial overview by providing a detailed examination of *how* the industry critiqued evidence supporting SP. We extend the current literature on TTCs' misuse and misrepresentation of science, summarised above, by building an in-depth conceptual account of industry arguments and practices used to undermine published evidence. We thus provide insights into the complexities of evidence-based policymaking where corporate interests conflict with public policy goals.

## Methods

### Data Sources

The four TTCs' submissions to the consultation were published in full on individual company websites. Our initial review of all four TTCs' submissions and coding of the volume, relevance, and quality of all the evidence cited by TTCs highlighted both the large volume of submissions and evidence cited. Submissions from the four TTCs totalled 1521 pages and cited 143 pieces of formal written research evidence [31]. We therefore limited our analysis to two companies, BAT and JTI [32],[33], chosen for the following reasons. BAT has a large market share in countries that have already implemented (Australia) or are considering (e.g., New Zealand) SP, making its strategy of wider interest. JTI has a large market share in the UK and Ireland (also considering SP), yet, unlike BAT and Philip Morris Ltd, was not subject to the litigation that led to document disclosure. With no internal documents available for scrutiny, far less is known about JTI, and analysis of its submission may therefore provide some insights into how the company seeks to influence policy.

We selected for analysis those sections of the BAT and JTI submissions (including expert reports) that focused on whether SP would produce the intended public health objectives (i.e., whether it would work). This represented a core topic in three (BAT, JTI, and PM) of the four TCCs' submissions and accounted for the largest volume of cited evidence (88/143) [31]. BAT and JTI also submitted a total of 14 expert reports covering different aspects of SP (two appended to the BAT submission and 12 submitted separately by JTI) [34],[35],[36],[37],[38],[39],[40],[41],[42],[43],[44],[45],[46],[47]. Of these, six (one BAT, five JTI) authored by three experts related to the core topic of whether SP would work. We selected the one relevant BAT report by Klick [34] and the two most recent JTI reports by Keegan and Devinney [38],[40] for analysis (Table 1).

**Table 1:** Number of pages analysed from all BAT and JTI submission documents.

Document type	Subject matter	Author, date	Number of pages	Number of pages analysed
British American Tobacco (BAT)				
*Main submission*	Consultation response	**BAT, 2012 ** [**32**]	**77**	**15**
*Expert reports*	Smoking attitudes and behaviours	**Klick, 2012 ** [**34**]	**15**	**4**
	Review of impact assessment	Gibson, 2012 [35]	30	0
All BAT documents			122	19
Japan Tobacco International (JTI)				
*Main submission*	Consultation response	**JTI, 2012 ** [**33**]	**116**	**26**
*Expert reports*	Smoking attitudes and behaviours	**Keegan, 2010 ** [**38**]	**105**	**29**
		**Devinney, 2012 ** [**40**]	**60**	**31**
		Keegan, 2008 [36]	90	0
		Keegan, 2009 [37]	35	0
		Devinney, 2010 [39]	125	0
	Smoking decision-making	Steinberg, 2010 [41]	98	0
		Dhar & Nowliss, 2010 [42]	60	0
	Economic impacts	Lilico, 2008 [43]	89	0
		Lilico, 2010 [44]	87	0
	Procedural: Better Regulation	Cave, 2010 [45]	61	0
	Trade agreements	Gervais, 2010 [46]	36	0
	Illicit trade	Chaudhry & Zimmerman, 2012 [47]	180	0
All JTI documents			1142	86
BAT and JTI documents (combined)			1264	105

Documents with pages analysed are indicated by **boldface**.

The reports by Keegan [38] and Devinney [40] critiqued a very large number of published studies on the impacts of SP, many of which were included in the Moodie review; Klick's report [34] was less detailed. We read all three [34],[38],[40] to form an idea of the type of critique offered, ascertaining that the critiques were similar, particularly those of Keegan, a business/marketing professor, and Devinney, an economics/business professor, although Devinney's tended to be more detailed. We therefore selected two of Devinney's study critiques for critical appraisal. Hammond et al, 2011 [48] was an online survey of young women (smokers and non-smokers) randomised to view different pack designs. The main finding was that fully branded female packs had greater appeal and were more likely to be associated with glamour, slimness, attractiveness, and less harm compared to plain packs. Thrasher et al, 2011 [49] conducted experimental auctions among adult smokers, and the main finding was that the lowest bid was associated with plain packs with prominent pictorial health warnings. We selected these study critiques because they contained detailed critiques using a wide variety of statistical and other methodological arguments that were also used for critiquing other studies.

### Analysis

We conducted four types of analysis. We examined the presentation of author CVs in all 14 expert reports. We then conducted two types of in-depth analysis on the selected documents (two main submissions and three expert reports): first, a verification-oriented cross-documentary analysis comparing references made to published sources with the original sources to ascertain how they had been used; second, an interpretive analysis to identify conceptual themes. The interpretive analysis was framed by the social constructivist perspectives of social studies of science [50],[51] and the premise that facts, including scientific facts, are socially and interactionally constructed and open to alternative interpretations. The literature on the industry's use and misuse of science (summarised in the Introduction) also formed a sensitising empirical backdrop to the analysis. We used thematic analysis informed by the approach and techniques of constructivist grounded theory [52],[53]: systematic conceptual coding (using *Atlas.ti* software), constant comparison, discourse sensitivity, attention to divergent data, and conceptual conclusions. One researcher (SU) read and micro-coded selected sections of the documents line-by-line for salient themes. Coding was inductive and emergent, although some themes replicated those identified during the preceding literature review, for example: (ignoring) weight of evidence; diversion (introducing alternative evidence); selective quoting; and wholesale discounting (of evidence). A sub-sample of the material (13%) was coded by two other researchers (JH, GF); there was considerable convergence in the initial analytic themes identified and agreement was reached through discussion on the differences in coding. The micro (first level) themes were grouped under broader categories; for example, the themes ‘misleading quoting’, ‘misleading interpretation’, and ‘selective quoting’ were grouped under ‘misleading quoting of evidence’. Emerging ideas were discussed by the wider team at interim analytic meetings. Finally, we conducted critical appraisal of Devinney's critique [40] of Hammond et al, 2011 [48] and Thrasher et al, 2011 [49]. Two authors (SU, AG) conducted this analysis with comments obtained from the original study authors.

## Results

The 15 pages (of 77) in the main BAT submission and 26 pages (of 116) in the JTI submission (41 of 193 pages (21%) combined) that dealt with the question of whether or not SP would work were analysed. Of the three expert reports we selected, we analysed 4 pages of Klick (BAT) [34], 29 pages of Keegan (JTI) [38], and 31 pages of Devinney (JTI) [40].

The BAT and JTI documents make the case that the evidence for SP was seriously and fatally flawed, delivering detailed and mostly externally commissioned critiques of the individual published studies and the Moodie review and reiterating that the DH had relied on ‘insufficient and unreliable’ [32] or ‘unreliable and unconvincing’ [33] evidence while ignoring other relevant evidence [32],[33]. We first look at the size of the submissions and their potentially debilitating effect on policymaking and then detail three industry practices—misleading quoting of evidence, mimicked scientific critique, and evidential landscaping—instrumental in undermining the evidence base for SP (Table 2).

**Table 2:** TTC practices for misusing scientific evidence, identified through analysis of TTC submission documents.

Type of Industry Practice	Techniques	
**Misleading quoting of evidence**	Misquoting	• Inaccurately reporting objectives, methods, findings, or conclusions of studies
	Selective quoting	• The ‘tweezers’ method: Reporting extracts out of context in a misleading way by partially quoting and/or omitting qualifying information
	Misinterpretation	• Presenting a minor point as a main conclusion• Presenting absence of evidence as evidence of absence
**Mimicked scientific critique**	Seeking methodological perfection	• Insistence on observation of actual behaviour• Exaggerating impact of limitations• Ignoring research governance
	Insisting on methodological uniformity	• Privileging marketing research• Rejecting qualitative methodology
	Adopting the litigation model	• Privileging experts• Piece-by-piece review
	Lack of rigour	• Incorrect reading/interpreting of studies• Double standards• Lack of clarity
**Evidential landscaping**	Promoting alternative evidence	• Citing behavioural studies of individuals to oppose a population-scale intervention
	Excluding relevant evidence	• Omitting internal industry research on the role of packaging in marketing

### Overwhelming Volume of Submissions

The size of the BAT (77 pages) and JTI (116 pages) submissions was their most immediately noticeable feature; the 12 expert reports that formed part of the JTI submission made up a further 1026 pages and the 2 in the BAT submission made up another 45 pages, bringing the total for the two companies to 1264 pages. In this study, it took one experienced full-time researcher 26 working days to analyse 105 pages, an average of 4 pages per day. If only half the 1521 pages submitted by four TTCs required close analysis, this would take 190 days (eight and a half months) to accomplish. Even a straightforward reading with no attempt at analysis would clearly take a very long time. Given that there were many other submissions from organisations and individuals (2444 in total, excluding brief ‘campaign’ responses [54]), this raises questions over the capacity of public bureaucracies to assimilate, synthesize, and consider the evidence submitted.

### Misleading Quoting of Evidence

This was a technique heavily used in the BAT and to a lesser extent in the JTI submissions. It involved inaccurate reporting of objectives, methods, findings, or conclusions of studies; presenting a minor point as a main conclusion; and the ‘tweezers method’ of partially quoting the original source and omitting qualifying information. The effect of all these practices was to distort or even contradict the meanings in the original source with the result that evidence supportive of SP was transformed into evidence against SP. There were 28 instances of this technique in the 15 pages analysed in the BAT submission and 13 instances in the 26 pages analysed in the JTI submission. Eliciting examples of this technique was labour and time intensive as it involved detailed examination of original sources. This technique relied for its effectiveness on the reluctance and/or inability of third parties (bureaucrats, interest groups, academic researchers) to devote time to such scrutiny.

A frequently employed method of inaccurate reporting was conflating absence of evidence with evidence of absence, i.e., arguing that no connection was found between smoking and packaging when the studies concerned had not explored this relationship. For example, in reporting results of a survey examining reasons for quitting [55], BAT claimed that ‘none of the main reasons stated involved packaging’ [32]. But examination of the survey reveals that packaging was not a response option, so it could not be selected as a reason. There were eight other instances of this technique in the BAT and JTI submissions.

BAT argued that, far from increasing the salience of health warnings, SP ‘may actually reduce smokers’ attention to warnings', citing as evidence a study in the Moodie review [56] that compared participants' eye movements when viewing branded and plain packs.

‘The researchers concluded that daily smokers exhibited more eye movements towards health warnings when the pack was branded than when it was plain…’ [32].

This was not the conclusion of the study by Munafo and colleagues [56]. The study concluded that SP had *no effect* on daily smokers whose attention to warnings remained the same (and did not decrease as suggested by BAT), while it appeared to *increase* attention to health warnings among non-smokers and light, non-established smokers.

BAT also claimed that the DH had previously concluded that there was not enough evidence to support SP:

‘When the Department of Health examined Plain Packaging … in 2008 it **concluded** that: “the research evidence into this [plain packaging] initiative is speculative, relying on asking people what they might do in a certain situation”’ [32, emphasis added].

This statement was not, however, a *conclusion*; the DH document listed five ‘positive effects’ and seven ‘potential disadvantages’ of SP, and the extract BAT quoted was one of the disadvantages [57].

One of JTI's criticisms of the Moodie review was that it had ‘inappropriately’ included studies ‘which exist only in abstract form’, something that would limit thorough critical assessment [33]. Three studies (out of 37) were cited as examples. Yet, despite the methodological challenges, it is considered good practice to include grey literature in systematic reviews in order to reduce publication bias [58],[59],[60]. Furthermore, the review's bibliography shows that for one [61], the authors had access to an associated PhD thesis and for another [62], to a draft/unpublished manuscript; it was not clear whether a full version was available in the third case [63].

JTI also claimed that the review's authors had ignored reports by two JTI experts, Devinney and Keegan, despite the citation of one by Devinney [39] in the review's protocol [64], resulting in the Moodie review being ‘at best flawed, and at worst, skewed’ [33]. Intriguingly, JTI acknowledged (in the same paragraph) that the reports could not have been included in the systematic review as they were secondary reviews and the systematic review only included primary studies. Furthermore, the Devinney report was cited in the Moodie review's protocol as an example of a review that ‘failed to adopt a systematic approach’, confirming the need for a *systematic* review [64], which JTI failed to mention when making its claim of exclusion.

While not exactly inaccurate reporting, the ‘tweezers method’ misrepresented original studies through selectivity. According to JTI, the Moodie review concluded that studies had failed to show that SP would have a positive impact on smoking behaviours:

‘… [the review] finds that the “overall pattern of findings is mixed”’ [33].

However, this sentence in the review continues: ‘…. mixed, but tends to be supportive of plain packaging being perceived to have a likely deterrent effect on smoking’ [30]. This part of the sentence was omitted from the JTI quotation.

BAT claimed that a US Food and Drug Administration (FDA) document confirmed that graphic warnings (which the Moodie review found would become more salient and effective on standardised packs) did not impact on smoking cessation:

‘… the US FDA Regulatory Impact Assessment estimated that the impact of the graphic warnings on reducing smoking rates was “in general not statistically distinguishable from zero”’ [33].

The FDA document in question relates to the introduction of graphic warnings in the US from September 2012 as a result of ‘… substantial evidence indicating that larger … warnings including a graphic component … would offer significant health benefits over the existing warnings’ [65]. The extract BAT quoted was from a technical appendix (p. 230 of the 231-page document) that explains the statistical uncertainty around the estimates. The full quotation is:

‘Although both of the estimation methods… lead to the conclusion that graphic warning labels will reduce smoking rates, FDA has had access to very small data sets, so our effectiveness estimates are in general not statistically distinguishable from zero; we therefore cannot reject, in a statistical sense, the possibility that the rule will not change the U.S. smoking rate. Therefore, the appropriate lower bound on benefits is zero’ (p. 230) [65].

The BAT submission quoted part of a report by the US Surgeon General [66] as corroboration of the company's argument that ‘packaging is not a relevant factor in driving [smoking]’ and that the ‘real’ drivers were parental and peer influences, risk preferences, socioeconomic factors, access, and price [32]. These factors are indeed highlighted in the Surgeon General's report [66] in Chapter 4, which covers ‘social, environmental, cognitive and genetic influences’ on smoking among youth and young adults. However, Chapter 5 of the report examines ‘the tobacco industry's influences on the use of tobacco among youth’ and concludes that there is ‘sufficient evidence’ of a causal relationship between ‘advertising and promotion’ and ‘initiation and progression’ of tobacco use (p. 10) and that there is ‘suggestive’ but not ‘sufficient’ evidence that tobacco companies have changed packaging and design in ways that have increased the products' ‘appeal to adolescents and young adults’ (p. 10). Chapter 5, in other words, suggests that packaging is a relevant factor in driving smoking, but this chapter is not mentioned by BAT.

### Mimicked Scientific Critique

This strategy, central to the JTI submission but also used by BAT, involved the detailed inspection, mainly by commissioned experts, of individual published SP studies and the Moodie review for methodological rigour and value, resulting in the rejection of the entire body of evidence for SP as flawed. This strategy worked at a deeper level because its discovery required broad epistemological understandings of science; specialised expertise in a large number of disciplines, research traditions, and methodologies; and skills in interpretive analysis, unlikely to be common among civil servants. Of the 37 studies in the Moodie review, 23 were subjected to parallel review across five JTI expert reports [36],[37],[38],[39],[40] (although, for two of these, unpublished versions were critiqued in industry reports). All 23 reported findings supportive of SP and all 23 were found to be ‘methodologically flawed and unreliable’; by contrast, in the Moodie review, 21 were rated as ‘medium’ and two as ‘high’ quality [30]. The industry experts found the Moodie review itself to be similarly flawed and unreliable. In reaching this conclusion, the experts appeared to use scientific methods of critique but close examination of the critiques showed that these were embedded in an essentially unscientific paradigm. They superficially resembled scientific peer review in that they declared the approach and evaluative criteria used in reviewing, identified methodologically problematic aspects of the studies (relating to design, data collection, and analysis) and explained how these might be overcome, and assessed the overall ‘reliability’ of the studies and whether their conclusions were justified by the reported findings. In doing so, the reviews used scientific terminology, even what might be called jargon. However, we identify and describe four features that marked these industry reviews as inauthentic and rendered them a ‘mimicked’ version of scientific critique: seeking methodological perfection; insisting on methodological uniformity; lack of rigour; and adopting the litigation model (Table 2).

#### Seeking methodological perfection

As SP is not widely implemented, observational evidence of its effects on smoking is not available. Research has therefore focused on three psycho-social *mechanisms* through which SP is likely to reduce smoking rates: reducing the appeal of packs and products; increasing the salience and effectiveness of warnings; and reducing misperceptions of product strength and harm [30]. This approach—obtaining the best available evidence within contextual limitations—is a key feature of scientific research [67],[68] but was vigorously rejected by the industry and its experts. BAT argued that mechanisms could only be considered *once* there was evidence of an actual impact on smoking behaviours, failing to acknowledge that the reverse was just as legitimate a scientific objective. JTI similarly rejected the legitimacy and value of mechanisms research.

Absence of ‘real world evidence’ [34], ‘actual behavioural outcomes’ [40], and correspondence between experimental results and ‘actual consumer behaviour’ [39] were repeated criticisms in industry experts' critiques of the SP literature. Studies were dismissed because they did not examine actual cigarette purchasing and smoking decisions or because experimental tasks did not replicate the real world. For example, experiments conducted in an environment where not all packages were plain could not be used to predict purchasing behaviours under a SP regime when all packaging would be plain [34],[38],[40]. Clearly, experiments are, by their nature, artificial and are often conducted because examining real-life behaviours and events is not feasible.

Because observational evidence cannot be obtained without the introduction of SP (as JTI acknowledged in its submission) [33], JTI, BAT, and industry experts effectively locked themselves into a nihilistic position that could potentially prevent the introduction of SP indefinitely: SP could not be introduced without evidence that it changed actual smoking behaviours; this evidence could not be obtained without the introduction of SP; and since SP could not be introduced without this evidence, SP could never be introduced. This premise was also future-proofed against emerging evidence from the Australian experience with SP:

‘… even if [SP legislation in Australia] survives legal challenges … differences between markets may confound comparisons or the transposition of effects’ [33].

It was suggested that the ‘ideal’ SP study, in line with ‘proper research design’, would be a randomised controlled trial that randomised different cities over a ‘long time period’ to having/not having SP [34]. This is clearly an unfeasible proposition not least because population mobility would mean that *both* groups have access to branded and standardised packages, rendering the results meaningless.

Perfectionism was also evident in the industry's representation of research limitations: the presence of limitations (regardless of nature, severity, and the extent to which they impact on the validity of findings) indicated wholesale failure of a study. All studies, including the best designed and conducted, are subject to some limitations, but one industry expert claimed that it was possible to design and conduct research with *no* limitations. While acknowledging that ‘no study was perfect’, he declared that ‘a well designed and executed study can avoid and/or correct for such limitations’ [38]. Having built this argument, the industry experts single-mindedly focused on identifying every conceivable limitation of the studies they reviewed (using narrowly defined criteria as explained in the following section, Insisting on methodological uniformity), while remaining silent on their strengths (Tables 3 and 4). Consequently, not one of the studies reviewed was found to provide ‘reliable’ evidence that SP would be effective [40], despite the vast majority having been published in peer-reviewed journals. Even one study that most closely fitted the experts' model of research and met virtually all their criteria, that by Thrasher and colleagues [49], was dismissed as ‘unreliable’ (Table 4).

**Table 3:** Industry critique of Hammond et al, 2011.

Hammond et al, 2011 [48]: On-line survey of 18- to19-year-old females (smokers and non-smokers) in the US; randomised exposure to different pack designs; rating for appeal and health risk+behavioural pack selection task. Finding: female branded packs associated with glamour, slimness, attractiveness, and less harm compared to plain packs.		
Devinney (2012) critique [40]	Review of Devinney critique	Practices of mimicked scientific critique
**‘the lack of actual behavioural outcomes or incentive compatible measures that represent how individuals would make choices in the broader context of purchasing are quite serious.’ (p. 31)**	The complexity of smoking uptake decisions and the fact that, for young people, cigarette packs may be proffered by friends rather than purchased appears not to be recognised. Nor does the fact that the outcomes were based on previous research (including tobacco industry market research) and specifically explore the mechanisms through which packaging (and SP) is likely to impact on smoking behaviour.	Insisting on methodological uniformity
	Behavioural task was used: respondents were asked which, if any, packs they would like to be sent upon conclusion of the study.	Lack of rigour
	Studying actual cigarette pack purchasing behaviour or any close incentive compatible proxy among young non-smokers (as included in the Hammond study) is likely to be deemed unethical.	Seeking methodological perfection
**‘The “Male brands” that are meant as controls have completely different brand names, pack dimensions and colours.’ (p. 34)**	In line with evidence cited by Devinnney that intentions most closely relate to actual purchasing when they are for existing products (p. 12), brands used are real brands. The reality is that male brands are different from female brands and thus the two cannot be both real and identical.	Lack of rigour
	The above is acknowledged in the paper and for this reason the male brands are excluded from some analyses.	Lack of rigour
**‘The research uses 5-point scales … these 5-point scales are then arbitrarily aggregated so that they are dichotomous (i.e., “1” and “0”). Such arbitrary aggregation is completely unacceptable based on the norms and standards of market research.’ (p. 32)**	This was not a market research study.	Insisting on methodological uniformity
	Dichotomous scales were not arbitrarily selected but used to provide a more intuitive metric. Furthermore, all analyses were repeated using both the five-point and the dichotomous outcome variable with the same pattern of results.	Lack of rigour
**‘Questions relating to “tar delivery” … and “health risks” are: (a) assuming that the individual is competent to understand the meaning of “tar delivery”; and (b) define “health risks” in a manner that is comparable between individuals…’ (p. 32). And later: ‘(a) it must be clear what is being rated – the “object” must be clear to those being asked to do the rating, (b) what makes up the construct is well articulated – that the “attributes” of the object are understood and valid, and (c) all of the raters are comparable – in other words, the raters are knowledgeable and relevant.’ (p. 33)**	Research shows most smokers and non-smokers have knowledge about the health risks of smoking and understand that tar is a toxin in cigarette smoke [92], [93].Decisions on tar delivery/health risks are made routinely when purchasing cigarettes without additional information being available to the purchaser. Provision of additional information in the study would introduce an artificiality that Devinney would seek to avoid.	Lack of rigour
	Tobacco companies have used similar measures in their own research.	Lack of rigour
	While it is possible that respondents had different conceptualisations of tar and health risk, etc., the differences will be balanced across experimental conditions given participant random allocation.	Lack of rigour
**‘The vast majority of the analyses are based upon pair-wise comparisons… where the scores are completely dependent upon the alternative against which they are being compared.’ (p. 35)**	Packages were rated individually, one at a time, and so the scores were not dependant on the comparator as suggested.	Lack of rigour
**‘The experimental conditions… do not allow for effective and efficient comparison of the package attributes, as the design is not efficient, orthogonal or balanced. For example, the brands appear different numbers of times … A properly designed study would control for brand effects, dimension effects, colour effects, price and other package and product attributes.’ (pp. 34–35)**	This fails to acknowledge the purpose of the study: to test the effect, on young women, of pack design (descriptors, colour and imagery) and of removing these elements (as would occur with SP). Had the intention been to study pack size, price, brand family, etc., and to determine which particular combination was more appealing (as might occur in a market research study), then these should have been balanced as suggested, but it was not.	Insisting on methodological uniformity
	Instead, and consistent with the study's objectives, the only differences between conditions were the elements being examined (pack design), while other elements (pack size and shape, brand family) were constant and the brands appeared an equal number of times across the three female experimental conditions.	Lack of rigour
**Various other complaints about the statistical analysis.** **e.g.: ‘Econometrically, the study has a number of flaws. Statistical efficiency would require that analysis of the preferences for specific brands be estimated using a pooled regression where the independent variables are conditional on the alternatives examined. In other words, because each participant will see a different mixture of eight packages we would want to know if the comparison set influences the choice. It is good practice to control for the choice set in which the evaluations are being made. Some bias might be mitigated by the ‘one at a time’ approach… but it is also likely that the mixture of items seen in the set of eight will have an influence. Normally good research practice is to control for items appearing in the evaluative set as a means of seeing whether any undue bias occurs.’ (p. 35)**	Incorrect interpretation of the study design and objectives as outlined above; all participants in each condition saw the same set of eight packages.	Lack of rigour
	While market research may have taken a different analytical approach, the critique fails to acknowledge that the between-group analysis in which participants are randomly allocated to groups is designed to reduce bias and that confounding was controlled for in the analysis.	Insisting on methodological uniformity
**Silent on study strengths**	Study strengths, including those consistent with the evaluative criteria set (Devinney p. 13–17) were: recent study; question design based on existing research; good sample size; subjects of relevant age (18–19 years); randomisation producing similar groups thus minimising bias; potential confounders controlled for; results statistically significant and consistent; included a behavioural task.	Seeking methodological perfection

**Table 4:** Industry critique of Thrasher et al, 2011.

Thrasher et al, 2011 [49]: Experimental auctions with adult smokers (US) bidding for different pack designs. Finding: lowest bid associated with plain pack with prominent pictorial health warning.		
Devinney (2012) critique [40]	Review of Devinney critique	Practices of mimicked scientific critique
**“Incompleteness in balance of design” & issues of bias: “as one moves from experimental condition 1 to 2 to 3 to 4 the severity of the warning gets higher…and this is obvious to all participants … individuals bidding on what is clearly a “superior “and “inferior” product … the study's intent was clear [to participants]” (p. 43)**	Each participant bid on two packs only, the second revealed only after bidding on the first. The order was randomised & shown to have no effect on bids, suggesting intent was not obvious.	Lack of rigour
	The study was not completely balanced in that sample sizes for the different attribute levels varied. This will have introduced some statistical inefficiency leading to less power to detect a significant result. This was not mentioned.	Lack of rigour
**‘The design lacks orthogonality. What would have been more appropriate would be to vary conditions orthogonally… What would then be discernible would be the true effects of each component independent of the others.’ (p. 43)**	Orthogonality is relevant where aim is to assess interactive effects of several components. Study aim was to assess impact of pictorial warnings & added impact of plain packaging, which was achieved.	Lack of rigour
**‘… the bidding task is … unrealistic to evaluate the importance of plain packaging. If plain packaging becomes the norm, the most appropriate case would be what happens when ALL brands are in plain packaging. Again, this could be incorporated into an experimental task by adding additional products into the bidding mixture. This solves two problems in the study. There is the fact that the study is clearly about cigarettes and health warnings on pack (as people see their preferred brand and two different packs). However, this can be hidden by having a basket of items on which they bid and the experimental design is the mixture of the types of products in the basket. One can then estimate the hedonic price of the various packs. In specific situations, as laid out in the experimental design, there will be plain pack versus plain pack alternatives, allowing the researcher to examine the price elasticity in very different market conditions.’ (pp. 43–44)**	Advantages/disadvantages of multiple experimental designs are subject to debate.	Insisting on methodological uniformity
	This argument is unclear, e.g.: is Devinney referring just to having cigarettes or also different products in plain packs in his intended design? If all brands are in plain packs, how will plain and ‘plain pack alternatives’ be compared?	Lack of rigour
**‘The bidding task is… unrealistic to evaluate the importance of plain packaging. If plain packaging becomes the norm, the most appropriate case would be what happens when ALL brands are in plain packaging.’ (p. 43)**	Impossible to replicate the real world	Seeking methodological perfection
	Study limitations acknowledged	Lack of rigour
	Fails to acknowledge that the experiment was close to real world (e.g., in grocery stores, real money used, smokers kept the packs), it is still useful in revealing the most likely demand estimate, and that limitations of the experiment were acknowledged.	Seeking methodological perfection
**Silent on study strengths**	Study strengths included: behavioural outcome; good sample size; randomisation; similar groups; statistically significant and consistent results. The study met many of the criteria set by Devinney, but this was overlooked.	Seeking methodological perfection

This view of limitations appeared to be operative in the industry's critique of the Moodie review, along with an apparent failure to understand the logic of synthesis:

‘Adding together multiple flawed studies does not make reliable an unreliable study … students who fail one exam do not have their position improved if they fail multiple exams’ [33].

‘Consistent results from studies that uniformly have the same methodological problems provide zero confidence in any conclusion except, perhaps, that the research designs were flawed in consistent ways’ [34].

In evidence synthesis, limitations of included studies are dealt with in two ways: studies judged to be below a quality threshold due to severe limitations are excluded; and the limitations of the included studies are taken into account through weighting and interpretation of results [58].

Perfectionism also led to suggestions that would breach research governance requirements. Devinney's insistence on studying actual purchasing outcomes or incentive-compatible measures [40], for example, would likely necessitate the exposure of young non-smokers (given that this is the target group of interest in standardised packaging) to attractive cigarette packs, a study design unlikely to receive ethical approval given what is already known on the impact of tobacco marketing (e.g., see Table 3). Keegan [38] and Devinney [40] argue that participants should not be informed of the purpose and sponsor of the research because this information would bias their responses. Failure to provide this information would breach ethics requirements in both social and health research, where researchers are obliged to inform potential participants of the purpose, methods, uses, and funding of the research [69],[70].

Demanding the perfect study design regardless of real-life and methodological constraints is clearly an unscientific position, at least outside the laboratory, but this was the premise of much industry critique of published SP studies, with the result that the reviewed evidence base for SP was discounted.

#### Insisting on methodological uniformity

The industry critiques of SP studies and the Moodie review were all framed by the discipline of market research and associated methodological conventions. The experts' disciplinary backgrounds included law, marketing, psychology, management, economics, and statistics; none declared expertise in tobacco control or public health. Keegan and Devinney reported using a set of assessment criteria for ‘good research’, citing mainly market research or public opinion research organisations as sources. Discrete choice experiments were designated as the best model for experiments involving purchasing decisions [40].These narrow standards were then used to systematically dismiss any study that did not fit the market research model, failing to recognise that other study types were valid and that many of the research objectives of the studies undertaken could not have been met via such a research model (Table 3).

Furthermore, the quantitative paradigm was accepted as the only scientifically acceptable one and qualitative methodology was dismissed, based on quantitative criteria, as useless.

‘… eight of the studies [in the Moodie review] were little more than small sample focus groups and two other studies were interview based; yet these were considered to be equally valid to other studies in the discussion’ [33].

‘Most of the normal checks that one attributes to good research practice … are violated [in focus groups]. One cannot control for truthfulness or incentive compatibility nor can the focus group questioning be subject to statistical analysis …’ [40].

In social science research, focus groups are not conducted to obtain statistical or generalisable findings but to elicit attitudes, beliefs, and experiences in a relatively naturalistic, informal manner [71]; their rigour is assessed using method-specific concepts and criteria. Qualitative studies, including focus groups, are increasingly recognised (e.g., by the UK's Cochrane Collaboration) as integral components in good quality evidence syntheses [72].

The Moodie review was dismissed as ‘the considered opinion of the author undertaking the review’ and ‘a focus group of researchers giving their opinion’ [33] because it used (qualitative) narrative synthesis rather than (quantitative) meta-analysis due to heterogeneity of the studies. Although challenging, narrative synthesis is an established method with authoritative guidelines available for its conduct [58]. The industry's criticism also appears to confuse a *systematic* review that uses narrative synthesis (e.g., the Moodie review) with a narrative, non-systematic, review [34].

The industry experts' methodological approach to reviewing the SP evidence base represents a fundamental failure to understand the requirement for methodological pluralism. A key indicator of quality in all research is the fit between the research question and the method(s) because no one paradigm or method can answer all types of research questions.

#### Lack of rigour

A central tenet of scientific review is diligence, and reviewers take great care to acquire a full and accurate understanding of the original sources they are reviewing; they use a disciplined and methodical approach, applying evaluative criteria consistently across studies to prevent biased judgment. In reviewing the two SP papers by Hammond et al [48] and Thrasher et al [49], JTI expert Devinney [40] appeared to disregard study objectives, experimental processes, analytic strategies, and steps taken by the original authors to minimise the impact of limitations on findings (Tables 3 and 4). In the main TTC submissions, as well as expert reviews, evaluative criteria were used inconsistently, leading in some instances to the use of double standards. For example, despite the industry's criticisms of self-reported beliefs, attitudes, experiences, and intentions in SP research, other studies that used these methods were promoted as good evidence because the results favoured the industry's case [32]. The TTCs criticised SP studies for jumping from data on particular populations to universal ‘speculations’ but this was exactly what the BAT submission did in arguing that SP might generate a ‘forbidden fruit’ effect and increase youth uptake of smoking [32]. This argument was based on a single study [73], the authors of which warned that its findings were ‘difficult to generalise and extrapolate’. The phenomenon of pack ‘appeal’, one mechanism linked to smoking uptake and therefore studied by public health researchers, was condemned as an ‘amorphous and vague concept … lacking in any evidential foundation … and arbitrary’ and could not justify the introduction of SP, JTI argued [33]. But JTI expert Devinney used the term liberally without objecting to it in his report [40] and Keegan referred to it in describing attitudinal research, for example to determine the appeal of detergent packaging [38].

One industry criticism of the Moodie review was that it was ‘inherently biased and self-interested’ [33] because its authors were ‘proponents and advocates of plain packaging’ [32] who worked together and ‘recycled’ information and methodologies [32]. This argument indicates a misunderstanding of scientific work. Evidence synthesis requires the collaboration of scientists/academics with a range of relevant expertise [58] working in the same or related fields and whose work is subject to peer-review. Furthermore, this criticism disregards the fact that the industry experts produced mainly single-author reports at the behest of the tobacco companies (although some declared independence) that were not peer-reviewed.

#### Adopting the litigation model

The industry reviews appeared to be embedded within a litigation—not scientific—model. Some industry experts referred to parts of their reports that would normally be labelled ‘appendix’ or ‘chapter’ as ‘exhibits’, and their critiques resembled courtroom testimonies aimed at demolishing the adversary's case. In the main TTC submissions, the experts were posited as sources of higher scientific authority, representing ‘the best contemporary scientific thinking’ [33] and were cited extensively. Demonstrating credibility was an important part of this project, with author CVs in the expert reports ranging between 10 and 20 pages and in one instance taking up 61 pages of a 98-page report [41]. Privileging the individual expert is a legal phenomenon and the legitimacy ascribed to individual experts' testimonies in the courts is fundamentally different from collectively established and consensus-based scientific legitimacy developed within specialised ‘communities of practice’ [74]. The tobacco company commissioned experts, working outside the peer-review system, dismissed the (peer-reviewed) evidence base for SP as flawed and unusable. In doing so, it was clear that they were attempting to establish an alternative system of scientific legitimacy.

Another manifestation of the litigation model was the experts' piece-by-piece approach to reviewing. Individual studies were examined in depth to determine whether any—on its own—constituted a warrant for SP and, following systematic deconstruction, none was found to be good enough to justify SP.

‘In summary … it is my expert opinion that none of the Studies provide [sic] reliable evidence that plain packaging would be effective in achieving the public policy goals of changing actual smoking behaviour …’ [40].

In court, each piece of evidence (i.e., each study and the Moodie review) is treated as a separate piece of evidence and each needs to be undermined and discredited in turn until no evidence remains that could damage one's client's case. By contrast, in scientific work, it is essential that the extant research is synthesised and greater confidence in the findings established through the cumulative ‘weight of the evidence’ [75].

To sum up, the two TTC submissions (by BAT and JTI) and the associated expert reports used different combinations of the techniques of mimicked critique we have documented here to dismiss the entire literature supportive of SP.

### Evidential Landscaping

As well as dismissing research that supported SP, another, more expansive, strategy in the submissions was to change the evidential landscape within which the policy debate was conducted by introducing and promoting research that examined non-packaging issues and, at the same time, excluding relevant industry research on packaging.

#### Promoting alternative evidence

The industry pointed to the literature on psychological and social explanations of smoking behaviours and individual decision-making to argue that packaging was irrelevant to smoking behaviours and decisions. The DH and the Moodie review were said to ‘ignore’ this parallel evidence base.

‘… the real drivers of smoking initiation include factors such as parental influences, risk preferences, peer influences, socioeconomic factors, access and price’ [32].

‘Numerous government funded and independent studies also show that factors other than packaging are the real drivers of decisions relating to quitting and relapse’ [33].

JTI submitted two expert reports to support this argument. One was prepared by two marketing professors who drew on the literatures on ‘consumer, cognitive and social psychology, behavioural economics and marketing research’ and concluded that SP would be ineffective because smoking decisions and behaviours were made on the basis of habit, goals and motives, peer influences, the consumer mindset, and self-control [42]. The other, by a psychology professor, argued changes in packaging did not affect ‘adolescents’ experimentation with or use of cigarettes', which were based instead on sensation-seeking, peer and family influences, a focus on rewards rather than risks, and availability [41]. While this body of research clearly makes an important contribution to knowledge on smoking behaviours, it overlooks the overwhelming evidence that tobacco marketing also plays a key role in shaping smoking behaviour [76] and cannot be legitimately used to argue that SP will prove to be an ineffective tobacco control measure. Smoking uptake is a complex phenomenon with multiple explanatory factors acting at both individual and population levels—only some of which are amenable to intervention. It is also a basic tenet of public health that population-level interventions, such as SP, by virtue of reaching the whole population, have a greater impact than individual-level interventions [77], yet, the majority of interventions promoted by the experts as more effective alternatives to SP were targeted at individuals.

#### Excluding relevant internal tobacco industry research

The DH consultation brief included a request for respondents to provide ‘any further evidence about tobacco packaging that you believe to be helpful’ [78]. Industry documents previously made public as a result of litigation and other more recent documentation [79] show that packaging is central to current marketing efforts and that the industry funds ‘systematic and extensive’ internal research on packaging [80]. This research covers RJ Reynolds (the international division of which was later taken over by JTI), Philip Morris, Brown & Williamson, American Tobacco Company, Lorillard Tobacco Company [80],[81],[82], and British American Tobacco Company [80],[82]. It shows that pack style can impact on smokers' perception of cigarette taste [80]; can (misleadingly) suggest that the contents carry lower health risks because they are portrayed as ‘milder’ or ‘lighter’ than other brands [82]; and is used by companies to increase product appeal to targeted groups, including young people [81],[82]. This body of evidence is therefore at least as—if not more—relevant to standardised packaging as the psycho-social body of evidence on smoking decision-making promoted by the TTCs in their submissions. It is clear both from this study and our earlier work [31] that none of the four TTCs referred to internal tobacco industry research on packaging in their submissions, despite citing a very large volume of other evidence to support their arguments. We do not know whether internal packaging research was shared with the DH on a confidential basis. However, the industry's public argument that packaging does not impact on smoking behaviours clearly lacks the benefit of the industry's own research on packaging.

## Discussion

We have reported the methods BAT and JTI used in their written submissions to undermine the evidence base for SP. The companies submitted a very large volume of evidence, reported studies in an inaccurate and misleading fashion, and sought to diminish the value and exaggerate the shortcomings of studies by using mimicked scientific critique. This last technique involved judging published studies against unrealistic and perfectionist criteria, dismissing research traditions and methods outside a narrowly defined paradigm (e.g., quantitative marketing research), adopting a litigation style that contrasted with the scientific model, and using non-rigorous review practices. In addition, they disregarded the consistency in the evidence for SP and tried to divert attention away from packaging to an alternative, less relevant body of evidence, while excluding a highly relevant body of industry research on packaging. These practices were conducted under the rubric of scientific critique and gave a misleading impression of scientific credibility. Our analysis suggests that they amount instead to an attempt to create a parallel and competing ‘scientific’ discourse. In formulating their response to the stakeholder consultation on SP, the tobacco companies reframed prevailing scientific norms and practices as somehow substandard and corrupt, seeking to impress on policy makers their own—distorted—interpretation of science and the scientific method.

It is not possible to ascertain how successful the tobacco industry was in influencing the UK government's stance on SP. Nevertheless, the government's announcement that it was postponing a decision due to concerns about the evidence base, combined with our findings and the industry's record in manipulating evidence to create policy inertia [83], suggest that industry plans to contest the evidence base [84],[85] met with some success, at least initially. The fact that this occurred despite the favourable conclusions of the Moodie review and strong evidence of the role of packaging in marketing [80],[81],[82] is notable.

This study had a number of limitations. Our analysis covered just two of the four tobacco company submissions. However, content analysis of how evidence was cited in all four submissions [31] suggests that our findings may be applicable to the other two TTCs, while leaked documents [84],[85] show Philip Morris International (PMI) also intended to contest the evidence base for SP. Additionally, we only focused on those sections dealing with whether SP would achieve the intended public health outcomes; we did not analyse sections on unintended consequences (economic, illicit trade, legal) that also cite and present different types of evidence. More generally, our research represents only one side of a dyadic phenomenon: the effectiveness of TTC attempts at influencing public health policy depends on how policymakers respond to such attempts. As intimated above, our current research is unable to examine this issue in any detail.

Nevertheless, our work provides crucial insights into how TTCs seek to discredit the evidence for a policy that runs counter to their interests and how regulatory tools associated with the Better Regulation agenda facilitate such efforts. In relation to the former, our findings are consistent with a broad body of literature on industry misuse of science and promotion of ‘sound science’. This literature shows that industries attempt to prevent or delay regulatory action aimed at limiting or removing harms associated with their products by creating doubt about the validity of scientific evidence that documents those harms. This extends from the tobacco industry [9],[10],[11],[13],[86] to dioxin (a synthetic carcinogen) [87], aspirin (in its association with Reye's syndrome), [12] and, more generally, to genetically modified corn (and its potential to spread in the wild) [88], acid rain, and global warming [8]. A recent study found that the alcohol industry adopted a similar strategy to the TTCs' in its submissions to the Scottish government's 2008 consultation on alcohol policies and minimum pricing [5]. So extensive is the practice of creating doubt and denying knowledge that Proctor has coined the term ‘agnotology’ (the cultural production of ignorance and its study) to describe, inter alia, the tobacco industry's manufacture of politically motivated ignorance in its attempt to transform evidential uncertainty into a paralysing search for certainty and subsequent policy inaction [83].

It is important to understand that the socially constructed nature of scientific work itself [50],[89] makes its manipulation by corporate interests possible. Science can be understood as a negotiated order sustained by craft conventions that are formulated, maintained, and altered over time within communities of practice. Methodological choices and compromises need to be made in response to contingencies; surrogate end points (e.g., mechanisms in SP research) are used when necessary; boundaries of acceptable limitations are defined; and different epistemologies (e.g., experimental, quantitative, and qualitative methods) are not only tolerated but put to productive use singly and in combination. Scientific knowledge is ‘simply the best knowledge available to a particular community working in a particular paradigm, with particular assumptions, instruments, and techniques’ [90]. This social nature of science constitutes a soft underbelly open to attack by commercial interests—tobacco and other—in their efforts to shape policy. A number of indicators have been suggested to help distinguish genuine scientific critique from the mimicked version. Two indicators suggested by de Camargo [91] are: ‘follow the money’, i.e., industry funding of the critique; and the intended audience, i.e., not scientific but public, legal, and regulatory communities. We concur with both. Our earlier work highlighted the significance of funding and independence [31]. For all the resources deployed by tobacco companies to undermine the SP evidence base, our analysis suggests that the industry's deconstruction was not particularly sophisticated from a scientific perspective. This may be because it was not intended to be persuasive to the scientific community, but was designed to speak to politicians, bureaucrats, and ultimately, in the context of judicial review, to judges. A third indicator, suggested by Jasanoff and again confirmed by our data, is disciplinary affiliation and perspective. Jasanoff points out that industry-commissioned experts, who ‘sow reasonable doubt about the reliability of particular scientific practices’, are often from different scientific ‘subcultures’ to those in which the original work was produced [92]. They use ‘idealised norms’ of scientific practice, deploy an ‘inherently unforgiving’ perspective, and their objective is to discredit by ‘exposing gaps and omissions’. Genuine scientific critique, on the other hand, is typically conducted by those located within the same scientific subculture (discipline/specialty) because only they ‘may be truly in a position to evaluate each other's competence’ and is aimed at improving the work so that it can contribute to ‘the larger enterprise of creating new knowledge’. Jasanoff's observation implies a fourth indicator—motivation. The TTCs' critiques were not intended, and did not originate, as contributions to the stock of scientific knowledge on SP but were produced in response to the public consultation on SP to support the TTC argument that SP should not be introduced.

While the tobacco industry has a long history of challenging scientific evidence in order to resist public health policies, the Better Regulation agenda, with its emphasis on evidence and requirement for stakeholder consultation, increases the utility of this strategy. This is something the TTCs recognise. Previous work shows that BAT predicted that the implementation of Better Regulation would enhance its ability to prevent public health policies [2]. Recently leaked PMI documents outlining the company's plans to prevent the introduction of SP in the UK suggest that PMI determined that ‘evidence based policy’, an invitation to ‘wait and see what happens in Australia’, and an emphasis on ‘Better Regulation’ should be central to its campaign along with the argument that SP would lead to increased illicit trade, adverse impacts on trade, and legal issues [84]. PMI also identified ‘evidence based argumentation’ as a strength of its corporate strategy in opposing SP and the prospect of ‘government ignoring Better Regulation principles’ as a threat to it [85]. The twin political imperative—to base policies and regulation on scientific evidence and, at the same time, to democratise science by inviting review from stakeholders—provides significant opportunities for tobacco and other corporate interests to challenge and obstruct regulation by challenging and undermining the evidence behind it. Questions therefore inevitably arise concerning the scientific value and public interest implications of subjecting evidence produced by non-commercially-affiliated academic researchers who have satisfied the increasingly elaborate and rigorous demands of public funding agencies, peer-review, and research governance systems to repeated episodes of review by those with a vested interest. Placing a formal obligation on civil servants to seek ‘stakeholders’ views on … key assumptions and data’ [3] arguably amounts to a vote of no or little confidence in the painstakingly constructed and expensive edifice of public research.

A further problem presented is the question of how such reviews are then handled by civil servants and politicians. Despite calls for transparency [93], the use of evidence in policymaking remains a black box. We do not know, for example, how closely the evidential critiques submitted by BAT and JTI were read and analysed and how much weight was placed on them. Without spending considerable time (given the volume of the TTC submissions) and without relevant scientific skills and knowledge, civil servants would have little choice but to take the industry's scientific critiques at face value. Alternatively, considerable public resources would need to be spent on analysis and verification by external experts.

## Conclusion

This study shows that the TTCs' critique of the evidence in favour of SP is highly misleading. Combined with our earlier work highlighting the very low quality of the TTCs' evidence against SP, it suggests that the TTCs' claim that SP will not lead to public health benefits is largely without foundation. It also highlights that the tools of Better Regulation, particularly stakeholder consultation, just as BAT predicted when pushing for their implementation, provide an opportunity for highly resourced corporations to slow, weaken, or prevent public health policies.

## Abbreviations

BAT: British American Tobacco
DH: Department of Health
SP: standardised packaging
JTI: Japan Tobacco International
PM: Philip Morris Limited
PMI: Philip Morris International
TTCs: transnational tobacco companies
